# DDT and Breast Cancer in Young Women: New Data on the Significance of Age at Exposure

**DOI:** 10.1289/ehp.10260

**Published:** 2007-07-24

**Authors:** Barbara A. Cohn, Mary S. Wolff, Piera M. Cirillo, Robert I. Sholtz

**Affiliations:** 1 Child Health and Development Studies, Center for Research on Women’s and Children’s Health, Public Health Institute, Berkeley, California, USA; 2 Mount Sinai School of Medicine, New York, New York, USA

**Keywords:** breast cancer, child health and development studies, exposure timing, *o*, *p*′-DDT, organochlorines, *p*, *p*′-DDE, *p*, *p*′-DDT, pregnancy, premenopausal

## Abstract

**Background:**

Previous studies of DDT and breast cancer assessed exposure later in life when the breast may not have been vulnerable, after most DDT had been eliminated, and after DDT had been banned.

**Objectives:**

We investigated whether DDT exposure in young women during the period of peak DDT use predicts breast cancer.

**Methods:**

We conducted a prospective, nested case–control study with a median time to diagnosis of 17 years using blood samples obtained from young women during 1959–1967. Subjects were members of the Child Health and Development Studies, Oakland, California, who provided blood samples 1–3 days after giving birth (mean age, 26 years). Cases (*n* = 129) developed breast cancer before the age of 50 years. Controls (*n* = 129) were matched to cases on birth year. Serum was assayed for *p,p*′*-*DDT, the active ingredient of DDT; *o,p*′*-*DDT, a low concentration contaminant; and *p,p*′*-*DDE, the most abundant *p,p*′*-*DDT metabolite.

**Results:**

High levels of serum *p,p*′*-*DDT predicted a statistically significant 5-fold increased risk of breast cancer among women who were born after 1931. These women were under 14 years of age in 1945, when DDT came into widespread use, and mostly under 20 years as DDT use peaked. Women who were not exposed to *p,p*′*-*DDT before 14 years of age showed no association between *p,p*′*-*DDT and breast cancer (*p* = 0.02 for difference by age).

**Conclusions:**

Exposure to *p,p*′*-*DDT early in life may increase breast cancer risk. Many U.S. women heavily exposed to DDT in childhood have not yet reached 50 years of age. The public health significance of DDT exposure in early life may be large.

Most previous studies do not support the hypothesis that exposure to DDT is an important risk factor for breast cancer (Lopez-Cervantes et al. 2004). However, previous studies were limited by the inability to measure exposure in young women during periods of the heaviest DDT use. Consequently most of these studies observed very low levels of *p,p*′*-*DDT [1,1,1-trichloro-2,2-bis(*p*-chlorophenyl)ethane] and *o,p*′*-*DDT [1,1,1-trichloro-2-(*p*-chlorophenyl)-2-(*o*-chloro-phenyl)ethane], the primary constituents of commercial DDT (Table 1). The conclusions of these studies apply to the effects of *p,p*′*-*DDE [1,1′-dichloro-2,2′-bis(*p*-chloro-phenyl)ethylene], the primary metabolite of *p,p*′*-*DDT (Lopez-Cervantes et al. 2004), which is more persistent in the environment and in biological systems and can therefore be measured years after DDT use had declined (Stehr-Green 1989).

In the present study we investigated whether serum *p,p*′*-*DDT and *o,p*′*-*DDT are associated with breast cancer, using blood samples obtained before DDT was banned and when use of this pesticide was very high (1960s). The median year of blood sampling in the present study was 1963, not long after the peak use of DDT in the United States in 1959 [U.S. Environmental Protection Agency (EPA) 1975] and near the peak dietary content of DDT estimated around 1965 (Wolff et al. 2005). Exposure declined considerably thereafter, even before the DDT ban in 1972 (Kutz et al. 1991).

This is the first study to measure blood levels in young adulthood (mean age of 26 years). In other studies, blood was collected when women were of middle age or older (Table 1). The present study is also the first study specifically designed, *a priori,* to consider whether age at exposure may modify DDT effects on breast cancer. Because DDT was first widely introduced in the United States beginning in 1945 (U. S. EPA 1975), a woman’s age in 1945 is a proxy for the youngest possible age at exposure to DDT and for her age when DDT use was peaking. A range of ages in 1945 is represented among women in the Child Health and Development Studies (CHDS). Moreover, these women could be observed prospectively. These data permit a unique design that tests the hypothesis that DDT associations with breast cancer are larger for birth cohorts in which women could have been most heavily exposed in early life.

## Materials and Methods

### Subjects

Subjects were participants in the CHDS, residents of the Oakland (California) area, and members of the Kaiser Permanente Health Plan who sought obstetric care between 1959 and 1967 (van den Berg et al. 1988). Subjects voluntarily participated in the CHDS, giving oral informed consent for an in-person interview, collection of blood specimens at several points during pregnancy and early postpartum, and permission for medical-record access. The present study was reviewed and approved by the Institutional Review Board of the Public Health Institute and, we have complied with all federal guidelines governing the use of human participants.

Breast cancer cases were identified by linkage to the California Cancer Registry and California Vital Status Records (Cohn et al. 2001). All names for each CHDS subject were submitted for cancer linkages using fixed (i.e., birth date, sex, race, and name) and changeable (i.e., address and patient record number) identifiers. A rigorous protocol was used to verify cases, comparing fixed versus changeable identifiers by manual review. The California Cancer Registry is reported to be > 99% complete after a lag time of about 2 years (Kwong et al. 2001).

Cases were defined as women with incident invasive or noninvasive breast cancer diagnosed before 50 years of age, or deaths due to breast cancer before age 50, obtained from linkage conducted in early 1998. A total of 133 cases met study criteria.

All members of the CHDS cohort are also linked to the California Department of Motor Vehicles (DMV) files on a regular basis to determine residence history, allowing us to assess their control status and to update any name changes. All names registered with the DMV were used in establishing a match. Simultaneous linkage of multiple family members enhanced matching. Regular DMV matching provides a history of location for each subject, which we used to determine the population at risk for cancer, corresponding with geographic surveillance by California’s cancer registries. Subjects who could not be located were considered lost to follow-up at the date of their last definitive classification as California residents. One control, matched exactly on birth year, was selected at random for each case from those who were under cancer surveillance and known to be free of breast cancer at the age of diagnosis for the matching case. The median time to diagnosis for cases was 17 years, and the mean age at diagnosis was 44 years.

### Serum assays

In 2000–2001, we measured DDT-related compounds in serum samples that had been collected during 1959–1967. The mean age of subjects when blood was drawn was 26 years. Samples collected within 1–3 days of delivery were used for 82% of the cases and 86% of controls, and serum drawn during the third trimester was used for the remainder. Longnecker et al. (1999), reported that organochlorine levels assayed across all trimesters of pregnancy and soon after delivery were highly correlated, indicating that the time when samples are collected over the 9 months of pregnancy is not critical. We stored serum samples at −20°C. Samples were first thawed to prepare an aliquot of 1.5 mL for organochlorine assays. The aliquots were shipped frozen to the laboratory; *p,p*′*-*DDE, *o,p*′*-*DDT, and *p,p*′*-*DDT were assayed using methods reported previously by Gammon et al. (2002). Sample order was randomly assigned within and across batches. Case–control pairs were analyzed in the same batches to minimize differences due to laboratory drift. The laboratory was blind as to case or control status of the samples. As described previously by Berkowitz et al. (2003), we used all observed positive values of *o,p*′*-*DDT in analyses, even those reported to be below the limit of detection (LOD); seven *o,p*′*-*DDT measurements with reported negative values were recoded as the lowest measured value (i.e., 0.01 μg/L). Interbatch and intrabatch coefficients of variation were 16% and 5%, respectively, for *p,p*′*-*DDT; 11% and 4% for *p,p*′*-*DDE; and 26% and 5% for *o,p*′*-*DDT. Total cholesterol and total triglycerides were measured enzymatically on a Hitachi 911 analyzer (Roche Diagnostics, Indianapolis, IN) in a laboratory certified by the Centers for Disease Control and Prevention (Atlanta, GA) and the National Heart Lung and Blood Institute Lipid Standardization Program (Bethesda, MD).

### Statistical analysis

Our results are based on 129 case–control pairs, matched on year of birth, after excluding 2 pairs with insufficient serum for lipid assays and 2 pairs with missing data on body mass index (BMI).

For individuals in 15 pairs for which the laboratory did not report data for *o,p*′*-*DDT, we imputed *o,p*′*-*DDT using analysis of covariance based on year of blood draw, number of prior pregnancies, breast-feeding, race, *p,p*′*-*DDT, and *p,p*′*-*DDE. Findings were similar when the 15 pairs with imputed *o,p*′*-*DDT were excluded from analyses (details available on request from authors). We present results where these 15 pairs are included.

We considered *p,p*′*-*DDT the primary analysis variable because it is the main constituent of commercial grade DDT. Initial analyses examined the effect of mutual adjustment for the three DDT-related compounds—*p,p*′*-*DDT, *o,p*′*-*DDT, and *p,p*′*-*DDE—where each compound was categorized in tertiles based on the control population and represented as two nominal variables: tertile 2 and tertile 3, where tertile 1 was the reference category. We performed data analyses using age-matched, conditional logistic regression. Breast cancer associations were compared for the following models: *a*) All three DDT-related compounds entered into the model simultaneously (model 1); *b*) compounds entered two at a time (model 2); and *c*) each compound entered alone (model 3). Based on the likelihood ratio test, we chose the best model for further examination of study hypotheses. Trend across tertiles of *p,p*′*-*DDT was tested using a continuous variable.

We examined whether age in 1945 (for the case–control pair) modified *p,p*′*-*DDT associations with breast cancer. This tested our *a priori* hypothesis that exposure to DDT in childhood and adolescence could increase susceptibility of the breast to DDT effects. Because DDT was first used widely in 1945 in the United States (U.S. EPA 1975), age in 1945 was used as a proxy for the youngest age when a woman could have been exposed. For example, women born before 1930 were not exposed during early adolescence, being > 15 years of age when DDT was first introduced. By 1959, the first year of CHDS study enrollment, when DDT use was at its highest, these women would be 29 years of age.

We coded age in 1945 as a four-category ordinal variable, defined by quartiles of age in 1945 represented in the study sample (< 4 years, 4–7 years, 8–13 years, and > 13 years). We estimated odds ratios (ORs) for *p,p*′*-*DDT tertiles within the age quartiles in 1945, adjusted for year of blood draw and for *o,p*′*-*DDT, coded as an ordinal variable representing tertiles of *o,p*′*-*DDT, coded at the median values in the control population (Greenland 1995); values were 0.22, 0.57, and 0.98 μg/L for tertiles 1, 2, and 3, respectively). *o,p*′*-*DDT was included in the model because it proved to be a confounder of the *p,p*′*-*DDT association.

To evaluate confounding by other measured breast cancer risk factors, we fit a series of models that entered one risk variable domain at a time to avoid adding a large number of variables to the model. Risk variable domains were race/ethnicity (African American, Asian, mixed race, with Caucasian as the reference category), number of previous pregnancies, blood lipids (total cholesterol and total triglycerides) (Longnecker et al. 2001), age at first pregnancy of ≥ 7 months, menarche before 12 years of age (yes or no), BMI [weight/ height^2^ (kilograms/square meter) measured at the first interview in early pregnancy (coded as two nominal variables: < 33rd percentile and > 66th percentile of the control distribution, or within the 33rd–66th percentiles), and whether the woman breast-fed after the observed pregnancy (yes or no).

## Results

All subjects had detectable levels of *p,p*′*-*DDE and *p,p*′*-*DDT (≥ 0.8 μg/L). Of the subjects, 65% had measurements of *o,p*′*-*DDT above the minimum detectable level of 0.4 μg/L.

**Figure 1:**
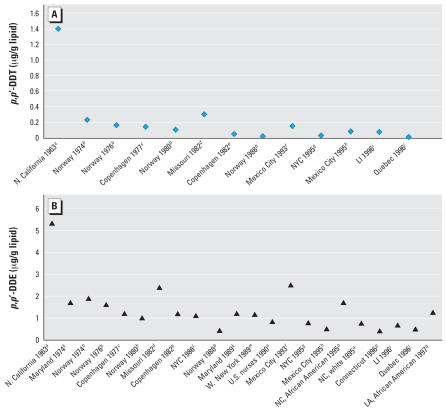
Reported *p,p*′*-*DDT (*A*) and *p,p*′*-*DDE (*B*) levels in blood observed in epidemiologic studies of breast cancer by year and place of blood draw (note the difference in scales). Abbreviations: LA, Los Angeles, CA; LI, Long Island, NY; N, northern; NC, North Carolina; NYC, New York City; W, western. Values shown are median, geometric mean, or arithmetic mean, depending on what was given in the original article. Only studies that reported lipid-adjusted levels in blood samples are included because lipids confound observed levels. ***a***Present study. ***b***Ward et al. (2000). ***c***Hoyer et al. (1998). ***d***Dorgan et al. (1999). ***e***Hoyer et al. (2000). ***f***Romieu et al. (2000). ***g***Wolff et al. (2000a). ***h***Lopez-Carillo et al. (1997). ***i***Gammon et al. (2002). ***j***Demers et al. (2000). ***k***Helzlsouer et al. (1999). ***l***Wolff et al. (2000b). ***m***Moysich et al. (1998). ***n***Hunter et al. (1997). ***o***Millikan et al. (2000). ***p***Zheng et al. (2000). ***q***Gatto et al. (2007).

Serum levels of *p,p*′*-*DDT and *p,p*′*-*DDE were considerably higher in the CHDS population than in populations where blood was sampled one to four decades later (Table 1, Figure 1).

At the time of blood draw (median year of 1963), all birth cohorts in the CHDS sample had been potentially exposed to DDT for roughly the same number of years (1945–1963). However, age at first possible exposure and age at blood sampling differs considerably among these women (Table 2). The age in 1945 quartiles represented in the CHDS population were < 4 years, 4–7 years, 8–13 years, and > 13 years (Table 2), but blood samples were drawn at a median age of 19 years for women who were youngest in 1945, compared with a median age of 36 years among women who were oldest in 1945 (Table 2). Both difference in age at exposure and age at blood sampling helps inform interpretation of findings for these four groups of women.

Table 3 presents estimates of breast cancer associations for DDT-related compounds for women in all age groups in 1945. *p,p*′*-*DDT was associated with increased risk of breast cancer, whereas *o,p*′*-*DDT was associated with a decreased risk of breast cancer. Adjustment for *o,p*′*-*DDT increased the *p,p*′*-*DDT association with breast cancer, but adjustment for *p,p*′*-*DDE made little contribution to association estimates for *p,p*′*-*DDT or *o,p*′*-*DDT, nor was *p,p*′*-*DDE significantly, independently associated with breast cancer.

Table 4 shows estimates of breast cancer associations for *p,p*′*-*DDT according to age in 1945, adjusted for *o,p*′*-*DDT and year of blood draw. There was an excess of *p,p*′*-*DDT in the serum of breast cancer cases a median of 17 years before diagnosis (*p* < 0.01 for linear trend; Table 4), but only among women potentially exposed before 14 years of age (*p* = 0.02 for DDT by age interaction; Table 4). BMI did not account for differences in *p,p*′*-*DDT associations by age in 1945, and we found no evidence that age in 1945 modified *o,p*′*-*DDT or *p,p*′*-*DDE associations with breast cancer (data not shown).

Table 5 presents associations for DDT-related compounds in women < 14 years of age in 1945, which can be compared with similar models for women of all ages shown in Table 3. Associations for *p,p*′*-*DDT were stronger in women who were < 14 years of age in 1945.

Table 6 presents a series of models that adjust *p,p*′*-*DDT and *o,p*′*-*DDT associations for other measured breast cancer risk factors in women who were < 14 years of age in 1945. There was little evidence of substantial confounding by any risk variables considered.

## Discussion

High levels of serum *p,p*′*-*DDT, a median of 17 years before diagnosis, predicted a 5-fold increased risk of breast cancer among women who were born after 1931. These women were < 14 years of age in 1945, the year when DDT came into widespread use in the United States. These women would have mostly been < 20 years of age as DDT use rose to its peak. Women who were not exposed to *p,p*′*-*DDT before age 14 (born in 1931 or earlier) and who would have been > 27 years of age when DDT use peaked, showed no increased risk of breast cancer according to serum levels of *p,p*′-DDT. There was no evidence that any adjustment variables examined, including BMI, explained the stronger *p,p*′*-*DDT association in women exposed at a young age.

Serum *o,p*′*-*DDT is one of the least persistent DDT-related compounds and is an indicator of recent, active exposure to DDT (Morgan and Roan 1975). Serum *o,p*′*-*DDT has usually not been studied in relation to breast cancer (Table 1). In the present study, we found that serum *o,p*′*-*DDT was inversely associated with breast cancer. This inverse association may be an indication that exposure to *p,p*′*-*DDT that occurred at a younger age, earlier in time, was the more important risk factor for breast cancer in this study population. On average, within *o,p*′*-*DDT tertiles, cases had higher levels of *p,p*′*-*DDT than their matched controls, as evidenced by the significant *p,p*′*-*DDT associations in models adjusted for *o,p*′*-*DDT (Tables 3, 5). We found no evidence that case–control differences in BMI could explain these findings (Table 6). Alternatively, the opposing direction of breast cancer associations for *p,p*′*-*DDT and *o,p*′*-*DDT could be explained by different metabolic pathways and hence varying exposures to intermediate products of metabolism. Metabolic studies have shown that the rate of metabolism of these two compounds differs, with *o,p*′*-*DDT eliminated more quickly (Morgan and Roan 1975).

Our results are consistent with the hypothesis that *p,p*′*-*DDT retained longer, possibly due to slower metabolism, is the underlying risk factor for breast cancer in this population. However, it is impossible to rule out an alternative explanation—that women at greatest risk were simply more heavily exposed at a critical age, some years before their blood was sampled or during their pregnancy.

Birth cohorts that did not show a *p,p*′*-*DDT association in this study were older when first exposed and also older when their blood was sampled (Table 2). We cannot distinguish between the significance of two factors: *a*) perhaps the earlier birth cohorts were not exposed at a vulnerable age; or *b*) perhaps we would have detected a *p,p*′*-*DDT effect in the earlier birth cohorts if we could have measured their exposure at a younger age. Thus, our findings do not rule out a *p,p*′*-*DDT association for earlier birth cohorts.

### Possible mechanisms

Direct toxicity of *p,p*′*-*DDT, induction of enzymes that produce other genotoxic intermediates and DNA adducts, or covariance with another as yet unknown factor are possible explanations of the associations we observed. Studies of polychlorinated biphenyls suggest that genetic differences in metabolism may interact with body burden to predict breast cancer risk (Moysich et al. 1999).

Genotoxicity is one possible mechanism for the *p,p*′*-*DDT association we observed. However, modern, highly sensitive molecular methods have only very recently been used to examine the genotoxicity of DDT in humans (Yanez et al. 2004).

Direct DNA damage to human blood cells, possibly with effects on immune surveillance, has received recent attention in studies of DDT exposure in Mexico, where DDT use was not banned until 2000 (Perez-Maldonado et al. 2005, 2006; Yanez et al. 2004). These authors paired *in vitro* investigations based on human blood cells with *in vivo* investigations of toxicity in blood samples collected from women and children in Mexico. Doses tested *in vitro* exceeded levels of DDT-related compounds observed *in vivo*. Nevertheless, *p,p*′*-*DDT and *p,p*′*-*DDE were associated with DNA damage *in vivo* for women (Yanez et al. 2004) and children (Perez-Maldonado et al. 2006), as well as *in vitro* (Yanez et al. 2004). Perez-Maldonado et al. (2005) suggested that *in vitro* studies do not accurately simulate *in vivo* conditions because humans are exposed chronically over a long period to mixtures of *p,p*′*-*DDT and its metabolites, including toxic metabolites other than *p,p*′*-*DDE. The median blood levels of DDT-related compounds we found in the present study were higher than average levels in women living in Mexico (Yanez et al. 2004). Also, the biological significance of DNA damage associated with DDT-related compounds is not clear (Yanez et al. 2004). Further consideration of these exposures in experimental settings and in human populations could lead to better understanding of mechanisms for the associations we observed in the present study.

### Comparison with other studies

The contrast between findings in the present study compared with largely negative results of previous studies can be explained by differences in study design. There are several reasons for discrepancies between the present study and most others.

#### *p,p*′-DDE as a proxy for DDT

*p,p*′-DDE may not be an adequate proxy for exposure to DDT. Commercial grade DDT consists primarily of *p,p*′*-*DDT, as well as a low concentration of *o,p*′*-*DDT. Neither of these compounds are as persistent as *p,p*′*-*DDE, a highly lipophilic metabolite formed from *p,p*′*-*DDT (Morgan and Roan 1975; Stehr-Green 1989). Humans form *p,p*′*-*DDE from *p,p*′*-*DDT particularly during periods of active exposure to commercial DDT (Morgan and Roan 1975). However, humans also ingest *p,p*′*-*DDE directly, as it is present in foods containing animal fat and is more persistent than its parent compound (Longnecker et al. 1997). Thus, *p,p*′*-*DDE levels in human serum may not accurately reflect past exposure to *p,p*′*-*DDT, particularly when blood samples are obtained decades after exposure to *p,p*′*-*DDT. Moreover, individual differences in metabolism and body fatness may further complicate the interpretation of serum levels of *p,p*′*-*DDE in serum samples obtained long after direct exposure to DDT (Perry et al. 2005; Wolff and Anderson 1999; Wolff et al. 2005). There is prior empirical evidence that *p,p*′*-*DDT exposure may not be meaningfully approximated by *p,p*′*-*DDE in human serum. The ratio of these compounds in human serum is variable, and a higher level of *p,p*′*-*DDT for a given level of *p,p*′*-*DDE has been reported to be associated with adverse outcomes, including longer time to pregnancy in daughters exposed *in utero* (Cohn et al. 2003) and primary liver cancer (McGlynn et al. 2006).

#### Varied biologic activity

Various DDT-related compounds do not have the same biologic activity. The compound *p,p*′*-*DDE acts as an antiandrogen but not as an estrogen; *o,p*′*-*DDT acts as an extremely weak estrogen; and *p,p*′*-*DDT shows little or no androgenic or estrogenic activity (Kelce et al. 1995). Thus, it is not likely that the *p,p*′*-*DDT association that we observed is due to estrogenic or androgenic activity. Moreover, it is reasonable to expect that the effects of these three compounds differ. Few prior studies measured all three compounds or considered all three compounds simultaneously (Table 1).

#### Exposure at critical periods

Prior human studies have not measured exposure during critical periods of susceptibility (Birnbaum and Fenton 2003). For the human breast, the critical periods appear to be during fetal life, adolescence, and early reproductive life, particularly before the first full-term pregnancy. Radiation, an established environmental risk factor for breast cancer, increases breast cancer risk most strongly when exposures occur early in life (Howe and McLaughlin 1996). Atomic bomb survivors < 20 years of age had the greatest excess risk of breast cancer (Tokunaga et al. 1994). These findings are consistent with rodent studies which show that effects of environmental exposures depend on whether the exposure occurs during critical periods of mammary development (*in utero*, during puberty, or during pregnancy) (Fenton 2006).

#### Influence of blood-sampling year

The year of blood sampling may influence the strength of DDT associations with breast cancer. In nearly all earlier studies, blood samples were collected in the mid-1970s and most much later, well after exposure to *p,p*′*-*DDT or *o,p*′*-*DDT could be directly observed for most women (Table 1, Figure 1). Only one previous study was based on samples collected during years of heavier DDT use (the 1960s), but *p,p*′*-*DDT and *o,p*′*-*DDT were not measured (Krieger et al. 1994). That study reported no overall association between *p,p*′*-*DDE and breast cancer (Table 1), which is consistent with the present study.

The U.S. EPA (1975) estimated that the maximum use of DDT occurred in 1959, and dietary DDT is estimated to have peaked around 1965 (Wolff et al. 2005). In the Second National Health and Nutrition Examination Survey, conducted between 1976 and 1980, *o,p*′*-*DDT was detectable in only 0.4% of human serum samples, whereas *p,p*′*-*DDT was detectable in 37.5% of samples (Stehr-Green 1989). In contrast, *p,p*′*-*DDE was detectable in 99.5% of these samples. These survey data are consistent with early metabolic studies which reported that the rates of elimination for DDT-related compounds differ considerably. Humans eliminate *o,p*′*-*DDT most rapidly, followed by *p,p*′*-*DDT, and then *p,p*′*-*DDE (Morgan and Roan 1975).

#### Age at diagnosis

Most previous studies have included both premenopausal and post-menopausal cases (Table 1), but even studies that did stratify findings by age at diagnosis did not find significant breast cancer associations for *p,p*′*-*DDT or *p,p*′*-*DDE in younger or premenopausal women (Gammon et al. 2002; Lopez-Carillo et al. 1997; Romieu et al. 2000). We speculate that these studies share a common limitation, namely that reported levels of *p,p*′*-*DDT observed were much lower than those found in the present study (Table 1, Figure 1). Studies conducted with blood samples drawn in the 1970s and later could be more subject to misclassification of early life exposure because of sampling well after peak DDT exposure (Figure 1).

#### Age at blood sampling

Most prior studies were based on blood samples that were obtained from middle-aged or older women (Table 1), whereas in the present study blood samples were obtained at a mean age of 26 years. Accordingly, the failure to observe increased risk in earlier studies where *p,p*′*-*DDT was measured may be explained if the breast is vulnerable to the cancer-promoting effects of DDT only during early breast growth and development.

#### Age at exposure

We found that serum *p,p*′*-*DDT was associated with breast cancer only for women potentially exposed at a young age (before 14 years of age). These women would also have been mostly < 20 years of age when DDT use peaked. This finding is consistent with results obtained in studies of exposure to atomic bomb radiation, where excess risk of breast cancer was observed primarily in women who were young at the time of exposure (Tokunaga et al. 1994).

### Limitations of the present study

We were unable to sample serum serially by age to determine more precisely when the body burden of DDT-related compounds was acquired. However, our findings support the hypothesis that initial exposure to *p,p*′*-*DDT during a critical period in early life is more important for breast cancer development than chronic exposure to its metabolite, *p,p*′*-*DDE. As found in several earlier studies (e.g., Lopez-Cervantes et al. 2004), *p,p*′*-*DDE was not related to breast cancer in this study population.

We could not determine how women acquired their exposure to DDT. However, we had information on farm residence in early life for 70% of our subjects because this question was added to a later revision of the intake interview. Among those with available information, 78% of cases and 74% of controls reported no residence on a farm, suggesting that most DDT exposure occurred in the context of urban life, probably through diet and direct contact for insect control.

We lack information on risk factors between the time of the pregnancy we observed and the subsequent development of breast cancer; thus, we were not able to fully adjust for completed parity, lifetime lactation, or fluctuations in weight before or after the blood draw. However, we were able to adjust for BMI in early pregnancy, breast-feeding following the observed pregnancy, and age at first pregnancy (one of the strongest reproductive risk factors for breast cancer).

Lactation after the observed pregnancy, which we could measure, may have helped clear lipophilic DDT-related compounds acquired in early life and may be more relevant to our hypothesis. Lactation was not a risk factor for breast cancer, and any clearance of *p,p*′*-*DDT due to lactation after the observed pregnancy did not appear to confound the *p,p*′*-*DDT association with breast cancer (Table 6). However, breast-feeding was rare and short-term among women in this study; only 34% of women breast-fed, and among those who did, 60% breast-fed for < 4 months. In a previous study of postmenopausal breast cancer, Moysich et al. (1998) found evidence supporting a stronger association for body burden of organochlorines among parous women who had never lactated, suggesting that lactation could help protect the breast. We did not see this interaction. The difference could be that our study measured exposure at a much younger age in relation to premenopausal breast cancer, which may have been initiated earlier in life.

It is possible that our method for adjusting for serum lipids may be imperfect and could also result in some residual confounding. Unmeasured and unknown confounders remain an alternative explanation of our findings.

We suggest that the higher serum levels of DDT-related compounds observed in the present study (Figure 1) are due to blood collection during peak, active DDT exposure. We have supported this interpretation by citing reports of declines in DDT-related compounds in human samples as DDT use declined. However, it is possible that population differences in lactation history or other factors could contribute to the differences seen in Figure 1.

Our study is limited by a relatively small sample size, and replication will be difficult because populations with samples obtained from young women during active DDT use are scarce. Follow-up of populations in countries with more recent and heavy use of DDT could provide new information. Timely sample collection during active exposure, in a population with a wide range of exposure, may increase power to detect effects even in small studies. For example, a study of dioxin exposure based on 15 cases in a cohort of 981 women accidentally exposed in Seveso, Italy, reported a significant dioxin effect on breast cancer (Warner et al. 2002).

The sample size for the present study might be considered a possible explanation of negative findings. However, the strong and statistically significant effect observed for *p,p*′*-*DDT (OR 5.4; *p* < 0.01) is of more interest given the sample size. Although the effect estimate was not precise [95% confidence interval (CI), 1.7–17.1; Table 4], the public health significance of DDT exposure is potentially large, even if the effect is actually closer to the lower limit of the 95% CI. This is because of the ubiquitous nature of DDT exposure.

## Conclusion

It is too soon to decide that DDT exposure has little public health significance for breast cancer risk. We base this conclusion on *a*) the long latency of possible effects on breast cancer; *b*) the large numbers of women exposed worldwide; and *c*) the evidence that we provide here which suggests that women exposed at a young age may be most strongly affected. Women born in the late 1950s and 1960s who were heavily exposed when young have not yet reached 50 years of age, let alone the age of greatest breast cancer risk.

## Correction

In the original manuscript published online, a data point for New York City in 1986 was inadvertently omitted from Figure 1B. It has been corrected here.

## Figures and Tables

**Table 1:** Studies of blood levels of DDT-related compounds and breast cancer.

Year of blood draw	Place	Design	Age at blood draw (years)	Age at diagnosis or percent premenopausal	Cases: controls	*p,p*′*-*DDE association	*p,p*′*-*DDT association	*o,p*′*-*DDT association	*p,p*′*-*DDE (μg/g)	*p,p*′*-*DDT (μg/g)	*o,p*′*-*DDT (μg/g)
1963	N. California (CHDS; present study)	Prospective, median follow-up 17 years	26	100% < 50 years	129:129	None	↑	↓	5.1	1.4	0.06 65% > LOD
1967	N. California (Krieger et al. 1994)	Prospective, mean follow-up 14 years	45	20%	150:150	None	NR	NR	NR^a^	NR	NR
1974 or 1989	Maryland (Helzlsouer et al. 1999)	Prospective follow-up ≥ 10 years for 70%	20% ≤ 40 (1974); 2.9% ≤ 40 (1989)	NR	340:340	↓	NR	NR	“DDE” b 1.7 (1974) 1.2 (1989)	NR	NR
1979	Norway (Ward et al. 2000)	Prospective, mean follow-up 9 years	41	60% < 50 years	150:150	None	None	NR	1.9 (1974) 1.6 (1976) 1.0 (1980) 0.44 (1988)	0.23 (1974) 0.16 (1976) 0.10 (1980) 0.02 (1988)	NR
1977 or 1982	Copenhagen, Denmark (Hoyer et al. 1998, 2000)	Prospective, mean follow-up 8 years (1977) and 5 years (1982)	55 (1977) 60 (1982)	32% 16%	240:477 155:274	None	None (1977); (Hoyer et al.1998) ↑ (for average of 1977 and 1982) (Hoyer et al. 2000)	NR	1.2 (1977) 1.2 (1982)	0.14 (1977) 0.05 (1982)	20% > LOD (1977) (Hoyer et al. 1998)
1982	Missouri (Dorgan et al. 1999)	Prospective follow-up ≤ 3 years for half, > 3 to < 12 years for half	57	21%	105:208	None	None	NR	2.4	0.3	4% > LOD
1986	NYC 1986 (Wolff et al. 2000b)	Prospective, follow-up 3–9 years	54	44%	148:295	None	NR	NR	1.10	NR	NR
1989	W New York (Moysich et al. 1998)	Retrospective	41–85	0%	154:192	None	NR	NR	1.15 (Laden et al. 2001)	NR	NR
1990	U.S. nurses (Hunter et al.1997)	Prospective, maximum follow-up 3 years (Hunter et al.1997); extended to 5 years (Laden et al. 2001)	59	18% (Laden et al. 2001)	372:372	↓, NS (Hunter et al. 1997); None (Laden et al. 2001)	NR	NR	0.82	NR	NR
1993	Mexico City (Romieu et al. 2000)	Retrospective	48	47%	120:126	↑, more so for postmenopausal women	None	NR	2.51	0·23	NR
1995	NYC 1995 (Wolff et al. 2000a)	Retrospective	54	37%	175:181	None	None	NR	0.66	0.03	NR
1995	Mexico City (Lopez-Carillo et al. 1997)	Retrospective	~50	50%	141:141	↓ , NS	None	NR	0.51	0.08	3% > LOD
1995	North Carolina, African Americans (Millikan et al. 2000)	Retrospective	50 (both races)	51% (both races)	292:270	None;↑ among thinnest, NS	NR	NR	1.69	40% > LOD	1% > LOD
1995	North Carolina, whites (Millikan et al. 2000)	Retrospective	50 (both races)	51% (both races)	456:389	↓, NS	NR	NR	0.76	40% > LOD	1% > LOD
1996	Connecticut (Zheng et al. 2000)	Retrospective	30–80	17% ≤ 45 years	475:502	None	NR	NR	0.46	NR	NR
1996	Quebec (Demers et al. 2000)	Retrospective	53	NR	315:219 or 307	None	None		0.48	0.01	NR
1996	LI (Gammon et al. 2002)	Retrospective	24–96	41%	633:418	None	None	NR	0.65	0.07	NR
1997	LA, African Americans (Gatto et al. 2007)	Retrospective	49.7	35–64	381:335	None	NR	NR	1.25	NR	NR

Abbreviations: ↓, risk declines as DDT compound increases; ↑, risk increases as DDT compound increases; LA, Los Angeles, CA; LI, Long Island, NY; N, northern; NR, not reported; NS, not statistically significant; NYC, New York City; W, western. Only studies that report lipid-adjusted organochlorines are presented, with the exception of Krieger et al. (1994), which is the only other study conducted with blood samples drawn in the 1960s. Lipid-adjusted organochlorine levels are presented to account for differences in lipid levels for study populations. Median levels or geometric means for controls are shown when available; otherwise, arithmetic means are presented. Organochlorine levels are not age-adjusted, so some differences by study population could be due to age differences; most studies reported higher organochlorines in older women. For *o,p*′*-*DDT, most studies only report the percentage > LOD, except for the present study. The year of blood draw is the median or mean year, and sometimes represents a single year. The age at blood draw is the mean or median reported for cases. If it was not given or could not be estimated, then the range is shown. For the Quebec study (Demers et al. 2000), two sets of controls were given: the hospital-based controls and the population-based controls.

a Lipid-adjusted levels of *p,p*′*-*DDE are not available, and *p,p*′*-*DDT and *o,p*′*-*DDT were not measured. The arithmetic mean concentration of *p,p*′*-*DDE was 43 μg/L (Krieger et al. 1994) compared with the median of 46 μg/L in the present study (CHDS); values are highly comparable because both studies were based on blood samples drawn in the 1960s from N. California women enrolled in the Kaiser Permanente Health Plan.

b Helzlsouer et al. (1999) defined “DDE” as “*p,p*′*-*DDT + *o,p*′*-*DDT + *p,p*′*-*DDE” for the 1974 cohort, and as “*p,p*′*-*DDT + *p,p*′*-*DDE” for the 1989 cohort.

**Table 2:** Characteristics of study subjects (*n* = 129 case–control pairs matched on year of birth).

	Characteristics of controls and cases							
	33rd percentile		50th percentile		66th percentile		Difference within matched pairs (case – control)	
Variable and age (years) in 1945^a^	Controls	Cases	Controls	Cases	Controls	Cases	Mean	SE
*p,p*′-DDT (μg/L)								
< 4	6.3	9.2	10.9	10.8	13.4	13.1	0.1	1.6
4–7	6.9	7.7	8.4	10.0	13.5	15.8	3.1	2.1
8–13	7.0	8.3	9.4	10.6	12.0	17.4	3.2	2.2
≤ 13	7.0	8.7	9.1	10.6	12.9	14.8	2.1*	1.1
> 13	11.9	9.5	14.0	13.6	18.2	15.4	−2.0	3.2
*p,p*′-DDE (μg/L)								
< 4	33.4	37.9	39.2	44.4	54.3	53.4	2.5	6.5
4–7	34.2	36.3	47.7	48.2	62.5	56.4	2.4	7.1
8–13	29.4	33.8	38.7	40.3	51.4	55.0	4.5	7.2
≤ 13	32.7	36.4	40.7	44.7	54.3	55.0	3.1	4.0
> 13	42.4	36.9	52.8	48.9	61.9	55.7	−5.2	6.6
*o,p*′-DDT (μg/L)								
< 4	0.42	0.47	0.57	0.54	0.73	0.70	0.07	0.15
4–7	0.45	0.39	0.66	0.52	0.79	0.67	−0.07	0.19
8–13	0.42	0.36	0.56	0.50	0.69	0.66	0.05	0.14
≤ 13	0.42	0.39	0.57	0.52	0.74	0.67	0.02	0.09
> 13	0.51	0.39	0.67	0.51	0.84	0.74	−0.23	0.15
Year of blood draw								
< 4	1963	1963	1964	1964	1965	1965	0.0	0.4
4–7	1962	1961	1964	1962	1965	1964	−0.9	0.6
8–13	1961	1961	1962	1962	1963	1964	0.1	0.5
≤ 13	1962	1962	1963	1963	1965	1964	−0.2	0.3
> 13	1961	1960	1962	1961	1963	1962	−0.4	0.4
Age at blood draw (years)								
< 4	19	19	19	20	20	21	0.0	0.4
4–7	23	22	24	23	25	24	−0.9	0.6
8–13	27	28	29	28	29	29	0.1	0.5
≤ 13	21	21	24	23	26	25	−0.2	0.3
> 13	35	33	36	35	37	36	−0.4	0.4
Age at first pregnancy (years)								
< 4	18	18	19	19	20	20	0.1	0.5
4–7	21	20	21	21	24	22	−0.7	0.7
8–13	21	24	23	25	24	27	2.0**	1.0
≤ 13	20	20	21	21	23	23	0.5	0.4
> 13	22	23	26	26	29	27	−0.3	1.4
BMI (kg/m^2^)^b^								
< 4	21	21	22	22	25	24	−1.0	1.1
4–7	21	21	22	23	23	24	0.6	0.7
8–13	21	2	24	23	25	24	0.7	1.0
≤ 13	21	20	22	22	24	23	−0.1	0.6
> 13	21	22	23	23	25	24	−0.2	1.0
No. of previous pregnancies								
< 4	0	0	0	0	0	1	0.1	0.1
4–7	0	0	0	1	1	1	−0.1	0.2
8–13	0	0	1	1	2	1	−0.6**	0.2
≤ 13	0	0	0	0	1	1	−0.2*	0.1
> 13	2	2	2	2	3	2	−0.3	0.4

a Age in 1945 corresponds to the earliest possible age of exposure to DDT; categories of age in 1945 (< 4, 4–7, 8–13, and > 13 years) correspond to quartiles represented in the study sample.

b BMI is based on measured weight and height obtained at an interview conducted in early pregnancy.

* *p* < 0.10, and

** *p* < 0.05 for paired *t*-test for the hypothesis that the within-pair difference = 0.

**Table 3:** Associations between DDT-related compounds and breast cancer with and without mutual adjustment: women of all ages in 1945 (*n* = 258; 129 case–control pairs matched on year of birth).

Model/variables	OR^a^	95% CI	*p*-Value
Model with all compounds			
*p,p*′*-*DDT			
Tertile 1	1.0	—	—
Tertile 2	1.9	0.9–4.1	0.09
Tertile 3	2.9	1.1–8.0	0.04
*p,p*′*-*DDE			
Tertile 1	1.0	—	—
Tertile 2	1.3	0.6–2.7	0.48
Tertile 3	1.0	0.4–2.4	0.92
*o,p*′*-*DDT			
Tertile 1	1.0	—	—
Tertile 2	0.5	0.3–1.0	0.06
Tertile 3	0.4	0.2–0.8	0.02
Models with two compounds			
Model 1			
*p,p*′*-*DDT			
Tertile 1	1.0	—	—
Tertile 2	2.0	0.9–4.2	0.07
Tertile 3	3.0	1.3–6.8	0.01
*o,p*′*-*DDT			
Tertile 1	1.0	—	—
Tertile 2	0.5	0.3–1.0	0.05
Tertile 3	0.4	0.2–0.8	0.01
Model 2			
*p,p*′*-*DDT			
Tertile 1	1.0	—	—
Tertile 2	1.5	0.7–3.0	0.26
Tertile 3	2.0	0.8–5.0	0.14
*p,p*′*-*DDE			
Tertile 1	1.0	—	—
Tertile 2	1.1	0.6–2.3	0.72
Tertile 3	0.7	0.3–1.7	0.40
Model 3			
*p,p*′*-*DDE			
Tertile 1	1.0	—	—
Tertile 2	1.8	1.0–3.4	0.06
Tertile 3	1.6	0.8–3.4	0.22
*o,p*′*-*DDT			
Tertile 1	1.0	—	—
Tertile 2	0.6	0.3–1.1	0.11
Tertile 3	0.5	0.3–1.0	0.06
Unadjusted models			
*p,p*′*-*DDT only			
Tertile 1	1.0		—
Tertile 2	1.4	0.7–2.7	0.33
Tertile 3	1.6	0.8–3.0	0.18
*p,p*′*-*DDE only			
Tertile 1	1.0		—
Tertile 2	1.5	0.8–2.6	0.19
Tertile 3	1.1	0.6–2.0	0.85
*o,p*′*-*DDT only			
Tertile 1	1.0	—	—
Tertile 2	0.7	0.4–1.3	0.22
Tertile 3	0.6	0.4–1.1	0.12

CI, confidence interval.

a ORs were estimated by conditional logistic regression. *p,p*′*-*DDT was represented as two indicator variables, tertile 2 and tertile 3, where tertile 1 was the reference category (tertile 1, < 8.09 μg/L; tertile 2, 8.09–13.90 μg/L; tertile 3, > 13.90 μg/L). *o,p*′*-*DDT was represented as two indicator variables, tertile 2 and tertile 3, where tertile 1 was the reference category (tertile 1, ≤ 0.42 μg/L; tertile 2, 0.43–0.72 μg/L; tertile 3, > 0.72 μg/L). *p,p*′*-*DDE was represented as two indicator variables, tertile 2 and tertile 3, where tertile 1 was the reference category: tertile 1, ≤ 35.23 μg/L; tertile 2, > 35.23–58.49 μg/L; tertile 3, > 58.49 μg/L). No variables are included in the models other than those noted.

**Table 4:** *p,p*′*-*DDT association with breast cancer stratified by the age of each case–control pair in 1945, the year DDT became widely used in the United States.

	All ages		Age quartile 1 < 4 years		Age quartile 2 4–7 years		Age quartile 3 8–13 years		Age quartile 4 ≥ 14 years		Age quartiles 1–3 < 14 years	
*p,p*′*-*DDT level	OR	95% CI	OR	95% CI	OR	95% CI	OR	95% CI	OR	95% CI	OR	95% CI
Tertile1	1.0	—	1.0	—	1.0	—	1.0	—	1.0	—	1.0	—
Tertile 2	1.9*	0.9–4.0	7.0*	0.9–55.5	4.1	0.6–29.3	1.4	0.4–5.4	0.7	0.1–3.3	2.8**	1.1–6.8
Tertile 3	2.8**	1.2–6.7	11.5*	1.0–138.9	9.6	0.7–137.2	3.9	0.8–19.2	0.6	0.1–3.2	5.4#	1.7–17.1
*p*-Value for trend^a^												0.01
*p*-Value for interaction between *p,p*′ DDT and age in 1945^b^												0.02

CI, confidence interval. All age groups include 258 subjects (129 case–control pairs) matched on year of birth. Categories of age in 1945 correspond to age quartiles in this sample. Quartiles 1–4 consist of 34 case–control pairs, 29 case–control pairs, 33 case–control pairs, and 33 case–control pairs, respectively. Uneven numbers by quartile result from the age distribution in the sample. ORs were estimated by conditional logistic regression models within subsets shown, matched on year of birth. Models included year of blood draw; *p,p*′*-*DDT represented as two indicator variables, tertile 2 and tertile 3, where tertile 1 was the reference category based on the distribution in the control population (tertile 1, < 8.09 μg/L; tertile 2, 8.09–13.90 μg/L; tertile 3, > 13.90 μg/L); and, *o,p*′*-*DDT was represented as a three category ordinal variable based on tertiles of the control population and coded at tertile medians of the control population (0.22 μg/L, 0.57 μg/L, and 0.98 μg/L for tertiles 1, 2, and 3, respectively.

a Based on *p,p*′-DDT coded as a continuous variable in a conditional logistic model, adjusted as described above.

b Estimated by a product term between a dichotomous variable for age in 1945 (< 14 years vs. ≥ 14 years) and *p,p*′ DDT (continuous variable) in conditional logistic model adjusted as described above.

* *p* < 0.10.

** *p* < 0.05.

# *p* < 0.01.

**Table 5:** Associations between DDT-related compounds and breast cancer with and without mutual adjustment: women < 14 years of age in 1945 (*n* = 192, 96 case–control pairs matched on year of birth).

Model/variables	OR^a^	95% CI	*p-Value*
Model with all compounds			
*p,p*′*-*DDT			
Tertile 1	1.0	—	—
Tertile 2	2.5	1.0–6.3	0.05
Tertile 3	5.2	1.4–19.1	0.01
*p,p*′*-*DDE			
Tertile 1	1.0	—	—
Tertile 2	1.5	0.6–3.4	0.34
Tertile 3	0.9	0.3–3.0	0.90
*o,p*′*-*DDT			
Tertile 1	1.0	—	—
Tertile 2	0.5	0.2–1.2	0.13
Tertile 3	0.3	0.1–0.7	0.01
Models with two compounds			
Model 1			
*p,p*′*-*DDT			
Tertile 1	1.0	—	—
Tertile 2	2.6	1.1–6.4	0.04
Tertile 3	5.0	1.7–14.8	0.00
*o,p*′*-*DDT			
Tertile 1	1.0	—	—
Tertile 2	0.5	0.2–1.2	0.12
Tertile 3	0.3	0.1–0.7	0.01
Model 2			
*p,p*′*-*DDT			
Tertile 1	1.0	—	—
Tertile 2	1.7	0.8–3.8	0.18
Tertile 3	2.9	0.9–9.1	0.06
*p,p*′*-*DDE			
Tertile 1	1.0	—	—
Tertile 2	1.3	0.6–2.7	0.56
Tertile 3	0.6	0.2–1.7	0.32
Model 3			
*p,p*′*-*DDE			
Tertile 1	1.0	—	—
Tertile 2	2.2	1.0–4.8	0.04
Tertile 3	2.1	0.8–5.2	0.12
*o,p*′*-*DDT			
Tertile 1	1.0	—	—
Tertile 2	0.6	0.3–1.4	0.21
Tertile 3	0.4	0.2–1.0	0.06
Unadjusted models			
*p,p*′*-*DDT only			
Tertile 1	1.0	—	—
Tertile 2	1.5	0.7–3.2	0.25
Tertile 3	1.9	0.9–4.2	0.09
*p,p*′*-*DDE only			
Tertile 1	1.0	—	—
Tertile 2	1.7	0.9–3.5	0.12
Tertile 3	1.2	0.6–2.4	0.62
*o,p*′*-*DDT only			
Tertile 1	1.0	—	—
Tertile 2	0.8	0.4–1.7	0.59
Tertile 3	0.6	0.3–1.2	0.18

CI, confidence interval.

a ORs were estimated by conditional logistic regression. *p,p*′*-*DDT was represented as two indicator variables; tertile 2 and tertile 3, where tertile 1 was the reference category (tertile 1, < 8.09 μg/L; tertile 2, 8.09–13.90 μg/L; tertile 3, > 13.90 μg/L). *o,p*′*-*DDT was represented as two indicator variables, tertile 2 and tertile 3, where tertile 1 was the reference category (tertile 1, ≤ 0.42 μg/L; tertile 2, 0.43–0.72 μg/L; tertile 3, > 0.72 μg/L). *p,p*′*-*DDE was represented as two indicator variables, tertile 2 and tertile 3, where tertile 1 was the reference category (tertile 1, ≤ 35.23 μg/L; tertile 2, > 35.23–58.49 μg/L; tertile 3, > 58.49 μg/L). No variables are included in the models other than those noted.

**Table 6:** *p,p*′*-*DDT association with breast cancer before and after adjustment for other risk factors: women < 14 years of age in 1945.

Model/variables	OR	95% CI	*p*-Value
Model 1			
*p,p*′*-*DDT tertile 1	1.0	—	—
*p,p*′*-*DDT tertile 2	2.8	1.1–6.8	0.03
*p,p*′*-*DDT tertile 3	5.4	1.7–17.2	0.00
*o,p*′*-*DDT (tertile 3 vs. tertile 1)	0.3	0.1–0.7	0.00
Year of blood draw	1.0	0.8–1.2	0.97
Model 2			
*p,p*′*-*DDT tertile 1	1.0	—	—
*p,p*′*-*DDT tertile 2	2.7	1.1–6.8	0.03
*p,p*′*-*DDT tertile 3	5.4	1.7–17.3	0.00
*o,p*′*-*DDT (tertile 3 vs. tertile 1)	0.3	0.1–0.7	0.01
Year of blood draw	1.0	0.9–1.2	0.88
No. of previous pregnancies	0.8	0.5–1.2	0.23
Model 3			
*p,p*′*-*DDT tertile 1	1.0	—	—
*p,p*′*-*DDT tertile 2	3.0	1.2–7.4	0.02
*p,p*′*-*DDT tertile 3	6.7	1.9–24.1	0.00
*o,p*′*-*DDT (tertile 3 vs. tertile 1)	0.3	0.1–0.6	0.00
Year of blood draw	1.0	0.9–1.2	0.88
Total cholesterol (mg/dl)	1.0	1.0–1.0	0.53
Total triglycerides (mg/dl)	1.0	1.0–1.0	0.95
Model 4			
*p,p*′*-*DDT tertile 1	1.0	—	—
*p,p*′*-*DDT tertile 2	2.8	1.1–7.0	0.02
*p,p*′*-*DDT tertile 3	5.8	1.8–19.0	0.00
*o,p*′*-*DDT (tertile 3 vs. tertile 1)	0.3	0.1–0.7	0.00
Year of blood draw	1.0	0.8–1.2	1.00
BMI tertile 1^a^	1.3	0.6–2.8	0.58
BMI tertile 3^a^	1.2	0.5–2.5	0.70
Model 5			
*p,p*′*-*DDT tertile 1	1.0	—	—
*p,p*′*-*DDT tertile 2	2.7	1.1–6.6	0.04
*p,p*′*-*DDT tertile 3	5.4	1.7–17.7	0.01
*o,p*′*-*DDT (tertile 3 vs. tertile 1)	0.3	0.1–0.7	0.01
Year of blood draw	0.9	0.8–1.1	0.50
Age at first pregnancy (years)	1.1	1.0–1.2	0.18
Model 6			
*p,p*′*-*DDT tertile 1	1.0	—	—
*p,p*′*-*DDT tertile 2	2.8	1.1–7.1	0.03
*p,p*′*-*DDT tertile 3	5.4	1.7–17.1	0.00
*o,p*′*-*DDT (tertile 3 vs. tertile 1)	0.3	0.1–0.7	0.00
Year of blood draw	1.0	0.8–1.2	0.92
Menarche before age 12	1.1	0.6–2.3	0.74
Model 7			
*p,p*′*-*DDT tertile 1	1.0	—	—
*p,p*′*-*DDT tertile 2	3.1	1.2–8.2	0.02
*p,p*′*-*DDT tertile 3	7.3	2.1–26.0	0.00
*o,p*′*-*DDT (tertile 3 vs. tertile 1)	0.2	0.1–0.6	0.00
Year of blood draw	1.0	0.9–1.2	0.61
African American^b^	0.9	0.4–2.0	0.75
Asian^b^	0.1	0.0–1.3	0.08
Mixed race^b^	1.7	0.6–5.3	0.35
Model 8			
*p,p*′*-*DDT tertile 1	1.0	—	—
*p,p*′*-*DDT tertile 2	3.0	1.2–7.7	0.02
*p,p*′*-*DDT tertile 3	6.4	1.9–21.5	0.00
*o,p*′*-*DDT (tertile 3 vs. tertile 1)	0.3	0.1–0.7	0.00
Year of blood draw	1.0	0.8–1.2	0.96
Breast-feeding in observed pregnancy (yes vs. no)	1.4	0.8–2.8	0.27

CI, confidence interval. ORs were estimated by conditional logistic regression. Each model is based on 192 subjects representing 96 case–control pairs matched on year of birth. *p,p*′*-*DDT was represented as two indicator variables, tertile 2 and tertile 3 where tertile 1 was the reference category (tertile 1, < 8.09 μg/L; tertile 2, 8.09–13.90 μg/L; tertile 3, > 13.90 μg/L). *o,p*′*-*DDT was represented as a three-category ordinal variable based on tertiles in the control population (tertile 1, ≤ 0.42 μg/L; tertile 2, 0.43–0.72 μg/L; tertile 3, > 0.72 μg/L) and coded at the median value within each tertile (0.22 μg/L, 0.57 μg/L, and 0.98 μg/L for tertiles 1, 2, and 3, respectively). ORs for *o,p*′-DDT are given for difference between the median in the third tertile and the median in the first tertile (0.76 μg/L). Model 1 is also shown in Table 4 (right-hand column; age quartiles 1–3, < 14 years) and is provided here to allow comparison with estimates after adjustment for other breast cancer risk factors.

a Reference category is 33rd–66th percentiles of BMI (kg/m^2^). Percentiles are based on distribution of body mass in controls. The 33rd and 66th percentiles were defined as ≤ 21.23 kg/m^2^ and > 23.71 kg/m^2^, respectively.

b Reference category is Caucasian.
